# Energy, Structures, and Response Properties with a Fully Coupled QM/AMOEBA/ddCOSMO Implementation

**DOI:** 10.1021/acs.jctc.1c00555

**Published:** 2021-09-03

**Authors:** Michele Nottoli, Riccardo Nifosì, Benedetta Mennucci, Filippo Lipparini

**Affiliations:** † Dipartimento di Chimica e Chimica Industriale, Università di Pisa, Via G. Moruzzi 13, I-56124 Pisa, Italy; ‡ NEST, Istituto Nanoscienze-CNR and Scuola Normale Superiore, Piazza San Silvestro 12, I-56127 Pisa, Italy

## Abstract

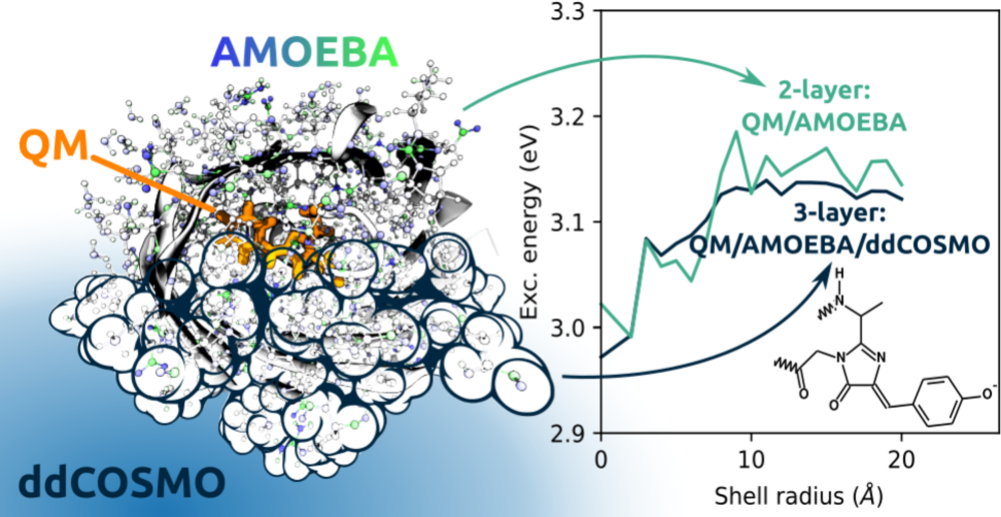

We present the implementation of a fully coupled polarizable QM/MM/continuum model based on the AMOEBA polarizable force field and the domain decomposition implementation of the conductor-like screening model. Energies, response properties, and analytical gradients with respect to both QM and MM nuclear positions are available, and a generic, atomistic cavity can be employed. The model is linear scaling in memory requirements and computational cost with respect to the number of classical atoms and is therefore suited to model large, complex systems. Using three variants of the green-fluorescent protein, we investigate the overall computational cost of such calculations and the effect of the continuum model on the convergence of the computed properties with respect to the size of the embedding. We also demonstrate the fundamental role of polarization effects by comparing polarizable and nonpolarizable embeddings to fully QM ones.

## 1 Introduction

In the years, multiscale models have acquired huge importance in the modeling of complex systems, and nowadays, they play a fundamental role in computational chemistry. Whenever the process under the study is localized on a specific region of the system and at the same time is tuned by the environment, the choice of describing the “active” part using an accurate quantum mechanical (QM) method and the rest of the system using a cheaper classical model has been shown to be very effective and reliable. Among the multiscale models, two main strategies have been widely applied. In the first approach, the environment retains its atomistic nature and each atom is represented with a particle, which behaves according to a simplified classical model, for instance, molecular mechanics (MM).^1−5^ In the second approach, instead, the environment is replaced with a featureless continuum, characterized only by a few macroscopic properties. This is the case of the polarizable continuum models (PCMs).^6−8^ Models of the first kind are particularly suited for the description of specific interactions (such as hydrogen bonds and coordinations) and anisotropic environments, such as the inner region of proteins. However, for proper modeling of the long-range electrostatic interactions, a large amount of MM atoms have to be included in the calculation.^9^ Models of the second kind, on the other hand, naturally take into account both long-range electrostatic effects and statistical sampling. However, their description of the specific short-range interactions is missing.

A possible strategy to further improve the modeling is carried out by combining atomistic and continuum approaches in a multilayered fashion. This strategy is promising because it benefits from the strengths of the two kind of models, allowing for a good description of the specific interactions and a cheap and simple description of the long-range electrostatic. The continuum model used for modeling of the outer shell must be particularly efficient for dealing with the increased number of atoms with respect to a standard QM/continuum calculation.

Several implementations have been proposed in the literature combining different nonpolarizable and polarizable MM formulations with different formulations of continuum models.^10−14^ In our research group, we combined an induced point dipole polarizable model with the integral equation formalism of PCM (IEF–PCM)^15^ and with the domain decomposition COSMO (ddCOSMO)^16,17^ for energies only.^18,19^

In this work, we go a step ahead with respect to these previous developments, by presenting a fully polarizable three-layer QM/MM/continuum approach, in which the atomistic MM shell is treated with the polarizable AMOEBA force field,^20,21^ and the continuum one with ddCOSMO. The present implementation is fully linear scaling in computational cost with respect to the number of MM atoms and includes energies, linear response properties, and analytical gradients. To the best of our knowledge, this is the first three-layer model that includes analytical gradients with respect to both QM and MM nuclear positions, using a cavity of molecular shape and scaling in a full linear way.

The paper is organized as follows. In Section 2, we derive the equations that define the three-layer model using a Lagrangian formalism. Then, we present the application to ground-state energy and forces within a self-consistent field formalism and to excitation energies and properties within the linear response approach, respectively.

In Section 3, we apply the newly implemented three-layer model to the study of geometries and excitation energies of three variants of the green fluorescent protein (GFP). These systems have been extensively studied using hybrid QM/MM approaches, which highlighted the sensitivity of their optical properties to short- and long-range electrostatic interactions.^22−26^ Here, we compare the three-layer model with a two-layer model on ground-state geometries and transition energies and dipoles. Moreover, we also investigate the effect of the polarizable force field by comparing a description based on AMOEBA with one based on a nonpolarizable force field.

## 2 Theory and Implementation

In this section, we derive the theory for the three-layer QM/AMOEBA/ddCOSMO model by deriving the equations for the energy and the energy gradients within a self-consistent field (SCF) QM framework.

A description of QM/ddCOSMO and QM/AMOEBA implementations can be found elsewhere.^27−29^ Here, it is sufficient to recall that AMOEBA is a force field, which models the electrostatics by endowing each MM atom with a set of fixed multipoles (charge *q*, dipole μ_s_, and quadrupole Θ) and an isotropic polarizability, the latter giving rise to the induced dipoles (μ_d_). The matrix, which defines the polarization linear system, is indicated with *T*. The embedding energy is computed as an interaction of the densities (*q*, μ_s_, Θ, μ_d_) with appropriate electrostatic properties of the QM density ρ.

On the other hand, ddCOSMO is a continuum solvation model, which solves the Poisson equation for a molecule placed in a cavity within a conductor. Given a representation of the solute’s electrostatic potential (Φ), ddCOSMO finds a representation of the reaction field due to the conductor (*X*), which can be used to compute the solvation energy as the electrostatic interaction between *X* and an appropriate function of the solute electrostatic density (Ψ). The ddCOSMO matrix is indicated with *L*. The quantities *X*, *L*, Φ, and Ψ are discretized over a basis of spherical harmonics with maximum angular momentum ; however, the electrostatic potential of the solute (*V*) is initially computed on a set of grid points, and only afterward, Φ is assembled using a numerical quadrature. The grid points are defined according to a Lebedev grid with *N*_grid_ points per sphere.

For the coupled case, we start the derivation from the general Lagrangian reported in ref (29). Considering that neither ddCOSMO nor AMOEBA is variational,^30^ the AMOEBA/ddCOSMO Lagrangian requires a total of six sets of degrees of freedom: the AMOEBA induced dipoles (μ_d_), the AMOEBA Lagrange multipliers (μ_p_), the ddCOSMO solution and Lagrange multiplier coupled with μ_d_ (respectively, *X*_d_ and *S*_d_), and finally those coupled with μ_p_ (respectively, *X*_p_ and *S*_p_).
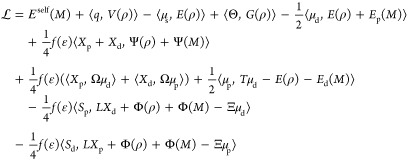
1The first term is the self-interaction of the AMOEBA fixed multipoles (where *M* stands for the collection of *q*, μ_s_, Θ). The three following terms are the interactions between the fixed multipoles and the QM density (ρ); *V*, *E*, and *G*, are respectively, the electrostatic potential, field and field gradient. The fifth term is the interaction of the induced dipoles with the QM and *M* densities. The sixth term is the ddCOSMO energy, i.e., the interaction of the reaction potential *X* with the QM and *M* densities, and the following one is the interaction between the induced densities of ddCOSMO (*X*_d_ and *X*_p_) and AMOEBA (μ_d_ and μ_p_). Finally, the last three terms are the constraints that enforce, respectively, the AMOEBA linear system (the term including the matrix *T*) and the ddCOSMO linear systems (for the d and p degrees of freedom of AMOEBA). The matrix Ξ, applied to the induced dipoles, represents their potential in the spherical harmonics basis used for ddCOSMO, and the matrix Ω, applied to the induced dipoles, is equivalent of computing Ψ(μ).

By differentiating eq 1 with respect to all the degrees of freedom, we get the coupled polarization equations. The first three coupled linear systems are obtained by imposing stationarity with respect to μ_d_, *X*_d_, and *S*_d_
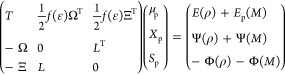
2The second set is obtained by imposing stationarity with respect to μ_p_, *X*_p_, and *S*_p_
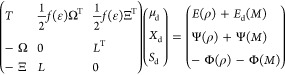
3

Once the linear systems are solved, in Lagrangian 1, many terms cancel out, and a simpler expression for the energy is obtained. We choose to simplify the two ddCOSMO constraints while leaving the AMOEBA constraint, as it results in a simpler expression. However, the latter constraint can be further rewritten so that it is easier to compute, by noting that *T*μ_d_ – *E*(ρ) – *E*_d_(*M*) = −1/2*f*(ϵ)(Ω^†^*X*_d_ + Ξ^†^*S*_d_). The expression for the energy thus reads
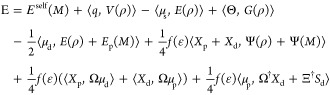
4

### 2.1 Energy and Linear Response

When coupled to a SCF method (either Hartree–Fock or density functional theory), a multiscale approach must provide at each QM iteration the embedding energy and its derivative with respect to density matrix elements, which is the contribution to the Fock (Kohn–Sham) matrix. The latter derivative can be obtained by differentiating the Lagrangian 1 with respect to a density matrix element *P*_μν_
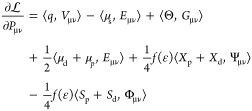
5In this expression, all the terms are contractions between a density and an electrostatic property due to the density of atomic orbitals μ, ν.

Algorithm 1 provides a schematic view of the steps required to perform an SCF calculation. At each QM iteration, given a QM density, first its electrostatic properties are computed and then the induced densities are found by solving the linear systems 2 and 3. Once these are found, the energy is computed using 4. Finally, the contributions to the Fock (Kohn–Sham) matrix are assembled according to eq 5.
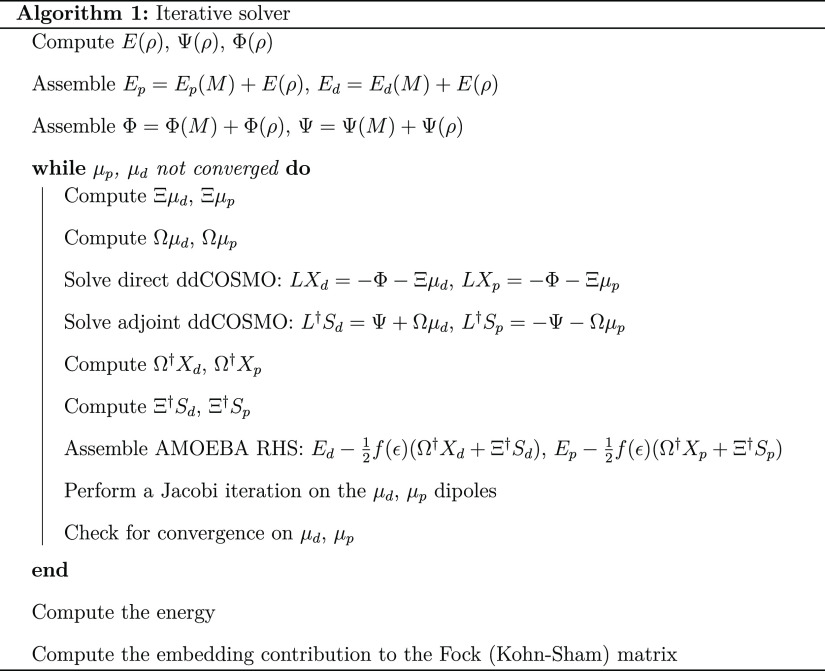


The QM/AMOEBA/ddCOSMO model has been extended to the description of excited states in the linear response formalism. The excitation energies and transition densities are found as the eigenvalues and eigenvectors of the Casida’s equations.^31^ In the case of a polarizable embedding, the orbital rotation Hessian is modified by a contribution which is the second derivative of Lagrangian 1 with respect to density matrix elements *P*_*ia*_ and *P*_*jb*_, where the indices run on occupied (*I* and *j*) and virtual (*a* and *b*) molecular orbitals. The second derivative reads
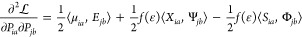
6In this expression, μ_*ia*_, *X*_*ia*_, and *S*_*ia*_ are the partial derivatives of the corresponding polarization densities induced with respect to the density matrix element *P*_*ia*_.

### 2.2 Forces

Once the ground-state calculation has been performed, and the ground-state QM density is available, the forces can be computed. Their expression can be derived by taking the gradient of Lagrangian 1. Here, it is convenient to distinguish between QM atoms (*R*) and MM atoms *r*
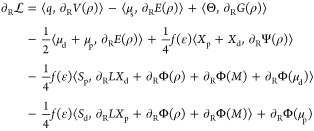
7
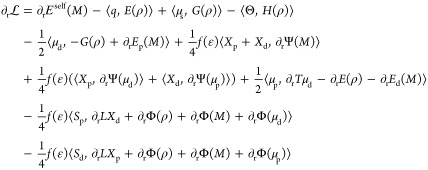
8In these expressions, several terms make up the plain QM/AMOEBA gradients, which have been discussed elsewhere.^28^ So we limit the discussion only to the novel contributions and the modified ddCOSMO gradient expressions.

For ddCOSMO, we recognize two kinds of terms, those involving a geometrical derivative and those involving a derivative of the RHS.^17,32^ For the first kind of contributions, the expressions are not modified with respect to a regular QM/ddCOSMO calculation: the cavity is now made of QM and MM spheres, but the geometrical relationships are the same and hence these terms are computed in the same way. The ∂Φ term is peculiar because it contains both the derivative of the potential and that of the characteristic function *U*. For this reason, it is convenient to split the two contributions into an electrostatic derivative Φ(*U*, *∂V*) and a geometrical derivative Φ(∂*U*, *V*), respectively. Using these considerations, eq 7 can be recast as
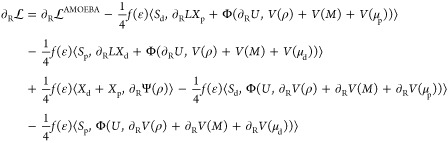
9In this expression, we recognize the QM/AMOEBA gradient ), the geometrical contributions to ddCOSMO gradients (second and third terms), the contribution from the gradient of Ψ, and finally two contributions from the gradient of the electrostatic potential (fifth and sixth terms).

The same considerations can be used to recast also eq 8 in a form that highlights the different kinds of contributions
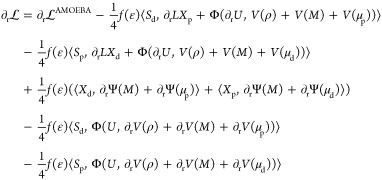
10The first term is the QM/AMOEBA gradient, and the second and third terms are the ddCOSMO geometrical contributions. The fourth term contains the derivative of Ψ, and finally the last two terms contain the derivative of the electrostatic potential.

The AMOEBA contributions to the gradients  are computed in the same way as in a QM/AMOEBA gradient calculation,^28^ and then, they are added to the total gradients. With respect to a standard QM/ddCOSMO implementation, there are a few technical differences concerning the geometrical contributions: in this case, there are two contractions (*S*_d_ with *X*_p_ and μ_p_ and *S*_p_ with *X*_d_ and μ_d_) instead of one (*S* with *X*), and the potential contains also contributions from the fixed multipoles and induced dipoles. As anticipated, the distinction between QM and MM spheres in this case is not relevant as the gradient terms are computed in the exact same way for both of them, as discussed in refs (17) and (32). Finally, contributions involving the gradients of Ψ and Φ require some extra steps and are discussed below.

The gradient of Ψ for the QM density has been already discussed in previous works,^27^ whereas the MM case is tackled as follows. First, we recall that the expression for Ψ at a given sphere *i* is a linear function of the multipoles at *i* and does not explicitly depend on the position of any sphere.^33^ For this reason, its gradient with respect to an MM atom is zero in the case of point charges and induced dipoles, as point charges and induced dipoles do not depend on atom positions. On the other hand, fixed dipoles and quadrupoles are obtained with a rotation operation, which translates them from a molecular frame (the one used in the parametrization) to a laboratory frame (the one used in the calculation). In this case, the rotation matrices depend on the position of the atoms, and hence, the contributions to the gradient are non-zero. A detailed derivation of the expression for the rotation matrices is provided in a previous work.^34^

Regarding the gradient of Φ, we recall that contractions in the form ⟨*S*, Φ(*U*, ∂*V*)⟩ are not evaluated in the ddCOSMO basis, but instead, they are recast as an interaction between pseudo-charges ξ at the grid points and the derivative of *V* at the grid points, so that it is possible to exploit the properties of the Coulomb kernel to ease the computation.
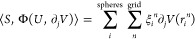
11Without going into details of the ddCOSMO theory, which can be found elsewhere,^27^ it is sufficient to recall that the pseudo-charges ξ depend on the quantity *S* and on geometrical parameters. The sum *i* runs on the spheres, whereas the sum *n* runs on the sphere-based grid points, *r*_*i*_^*n*^ is the position of grid point *n* on sphere *i*. With this premise, the gradient contributions of eqs 9 and 10 take the following form
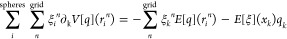
12where the notation *V*[*q*](*r*) means electrostatic potential from sources *q* computed at *r*, and *E* is the electric field. In this expression, the gradient is computed with respect to the atom *k* which can either be a QM or MM atom. In the former case, however, a fixed multipole at position *k* is missing and the second contribution is zero. A similar expression can be written also for the dipoles and the quadrupoles
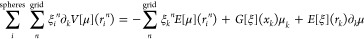
13
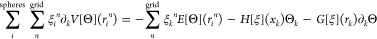
14where *G* and *H* are, respectively, the electrostatic field gradient and field hessian. With respect to eq 12, we note additional terms stemming from the rotation matrices containing the derivatives of the rotation matrices ∂_R_μ_s_ and ∂_R_Θ.^34^ Finally, we note that eq 13 holds also for the induced dipoles, but in that case, the term from the rotation matrices is missing.

### 2.3 Linear Scaling Implementation

The model was implemented into a locally modified version of the Gaussian 16 suite of programs,^35^ which, we recall, is also coupled to the Tinker package,^36,37^ so that we can perform multiscale molecular dynamics and geometry optimizations using the AMOEBA force field.^38−40^ The new implementation generalizes the previous QM/ddCOSMO and polarizable QM/MM implementations, so that the solute in the ddCOSMO calculation is made of both QM and MM atoms.

The implementation is based on three main driver routines, one for the SCF, one for the linear response, and one for the forces. Furthermore, it allows for a linear scaling computational cost by using the fast multipole method implementation presented in ref (41). The SCF code implements all the steps reported in algorithm 1. The linear response code is a modified version of the SCF routine: the linear response eigenvalue problem is solved using an iterative Davidson diagonalization which requires only performing matrix–vector products between the matrix defined in eq 6 and the transition density.^42^ The result of the matrix–vector product is equivalent to the Fock matrix element reported in eq 5, with the difference that in this case, the indices refer to molecular orbitals, the starting density is a transition density and there are no contributions from nuclei and fixed multipoles.

The implementation has to be able to deal with large systems, for instance, chromophores embedded in proteins with thousands of MM atoms, which requires its computational cost to scale linearly with respect to the number of MM atoms. To achieve a fully linear scaling implementation in the number of MM atoms, each step of the computation has to be implemented and formulated in a linear scaling fashion. This is the case for the solution of the ddCOSMO steps: thanks to the sparsity of *L* and to the use of an iterative solver, the ddCOSMO linear system can be solved in  operations. This is also the case for interactions between the QM atoms and the MM atoms or interactions between the QM atoms and the cavity points and for the computation of Ψ, its gradient, and the coupling terms Ωμ and Ω^†^*X*.

The remaining terms are in principle quadratically scaling in the number of MM atoms. In this case, the first step for achieving a linear scaling implementation is to rewrite these terms as either electrostatic properties at the MM atoms of a density defined on the MM atoms or as interactions between a target density and an electrostatic property of a source density (where both the source and target densities are defined on the MM atoms). Among the former terms, we have the computation of the right-hand sides [*E*_p_(*M*), *E*_d_(*M*), and Φ(*M*)], the coupling terms (Ξμ and Ξ^†^*S*), and the matrix–vector products involved in the dipole linear systems (*T*μ). Among the latter terms, we find the contractions appearing in the energy expression 4 and in the forces expressions eqs 12–14.

As an example, we report here the steps required to transform contributions Ξμ and Ξ^†^*S* into the computation of an electrostatic property of some charge distribution, which can be accelerated using the fast multipole method. The definition of a matrix element of Ξ is as follows
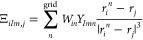
15where  and *m* are the spherical harmonic indexes, *i* is the index of the spheres, *j* is the index of the dipoles, *W*_*in*_ are the weights associated with the numerical quadrature,  is a given spherical harmonic function evaluated at point *n*, finally *r*_*i*_^*n*^ and *r*_*j*_ are, respectively, the positions of grid point *n* on sphere *i* and of dipole *j*. Because the algorithm does not require the explicit matrix, but only to evaluate its effect on *S* and μ, it is possible to reorder the sums in such a way that an electrostatic property appears. For the direct action of the matrix, we write
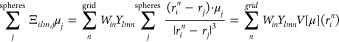
16where we recognize the potential of all the dipoles *V*[μ] which has to be computed at all the grid points and in principle is a  scaling contribution. Once this quantity is assembled, the remaining numerical quadrature can be performed in a  step. For the action of the transpose, instead, we write
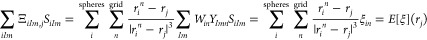
17Also, in this case, the effect of the matrix is rewritten as a two-step process: first the pseudo-charges ξ_in_ are assembled in a  step, and then, the field of ξ at all the targets *r*_*j*_ has to be computed, which is a  step.

Once all the  steps have been rewritten in terms of an electrostatic property of a given density, the fast multiple method can be employed to reduce the computational cost associated with the computation of the electrostatic property. In this work, we adapted the previous implementation^41^ to handle also the computation of electrostatic properties of densities defined on the cavity points at the MM atoms and the properties of densities defined on the MM atoms at the cavity points. We achieve therefore linear scaling in all the operations related to the embedding for the computation of both energies and forces.

## 3 Application to GFPs

**Figure 1:**
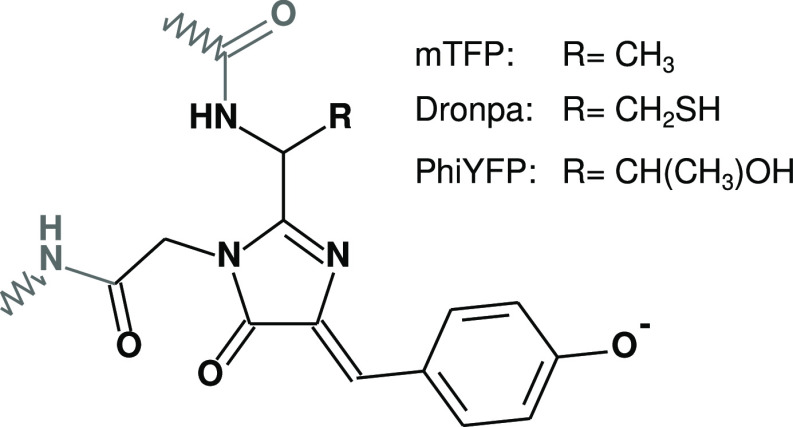
Structure of the chromophores for the investigated systems. The parts in gray depict the atoms of the linked residues, which have been added to the QM part.

The QM/AMOEBA/ddCOSMO model presented in the previous sections was applied to the study of the absorption energies of three different variants of the GFP, namely, the mTFP0.7 (hereafter **mTFP**), **Dronpa**, and **PhiYFP** proteins. In all these systems, the chromophore is a modified tyrosate residue condensed with the two adjacent residues, namely, a glycine residue and another residue specific to the GFP variant: alanine for **mTFP**, cysteine for **Dronpa**, and threonine for **PhiYFP**. A representation is provided in Figure 1. The different structure of the chromophore combined with a different local environment tune the spectroscopic properties of the three GFP variants.^43−46^ The chosen GFP variants span the broad variation of excitation energy of GFP-like fluorescent proteins containing the same 4-(*p*-hydroxybenzylidene)-5-imidazolinone chromophore in the anionic protonation state: **mTFP** and **PhiYFP** are among, respectively, the most blue-shifted and most red-shifted variants, while **Dronpa** is the intermediate between the two. The measured excitation energies of 2.74, 2.46, and 2.36 eV are experimentally observed for **mTFP**,^47^**Dronpa**,^48^ and **PhiYFP**,^49^ respectively.

To test the newly proposed QM/AMOEBA/ddCOSMO model, we performed a series of calculations on systems of increasing size for the three GFP variants and compared them against different models.

In the following sections, we use the term three-layer to indicate the QM/AMOEBA/ddCOSMO model and two-layer to indicate the QM/AMOEBA model.

### 3.1 Computational Details

**Figure 2:**
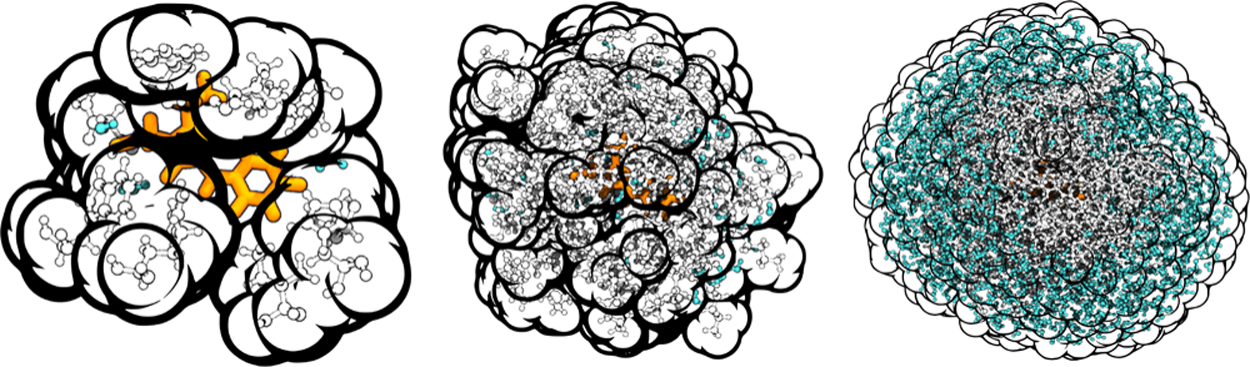
Representations of three **mTFP** structures with shells of different radii (2.3, 9.8, and 22.1 Å). The QM part is represented in orange, the MM protein in white, and the water molecules in cyan. The cavity is schematically drawn.

As a starting point, we used the structures from a previous work.^25^ They are obtained from protein data bank structures 2OTB,^47^ 2Z1O,^50^ and 4HE4^49^ for **mTFP**, **Dronpa**, and **PhiYFP**, respectively. The proteins are solvated in a water box (a 75 Å truncated octahedron containing about 9500 water molecules) with Na^+^ and Cl^–^ ions in a 0.1 M concentration, with an excess of Na^+^ to neutralize the negative charges of the proteins. From these, we generated systems of increasing size by selecting the chromophore and all the residues within fixed distances from it. Given two residues we used the minimum distance between them as a distance definition. Two different strategies were used for the generation of the systems: in one strategy, we selected shell radii from 2 to 25 Å evenly spaced by 1 Å, thus obtaining both neutral and charged structures, whereas in the other strategy, we selected only shell radii that allowed for a total net charge of zero (we recall that the QM part is negatively charged). Supporting Information provides a more detailed description about the resulting neutral structures: a table summarizes the shell radii, the number of atoms in the MM shells, and the fraction of protein residues included in a given shell, and a figure provides a graphical representation of the smallest neutral structures for the three systems. Figure 2 shows three example structures for the **mTFP** system.

In all cases, the QM subsystem included the chromophore and the carbonyl and N–H moieties of the linked amino acids (Figure 1). A link atom scheme was used to account for the QM–MM covalent bonds, by placing a hydrogen atom along the QM–MM bond at 1.0 Å from the QM heavy atom. Overpolarization of the QM density was prevented by removing the electrostatic parameters from MM atoms in position 1, 2 and 1, 3 with respect to the QM atom at the interface. The total charge of the cropped MM residue was then set to the appropriate integer value by redistributing the excess or defect charge over the remaining atoms of the residue itself.

The MM part was either described using the AMOEBA polarizable force field^21^ or the AMBER ff99SB nonpolarizable force field.^51^ In all the cases in which a bond between two amino acids was cut by the cropping scheme, we did not employ any special measure for the amino acid termination.

For the external ddCOSMO solvation shell, we used a static dielectric constant of 15.0 and an optical dielectric constant of 2.0. The static dielectric constant is chosen to be in between the typical values for the interior and for the exterior of the proteins.^52^ The boundary between the QM/MM and the continuum solvent was defined using a solvent accessible surface. For ddCOSMO, we used  = 6 and *N*_grid_ = 110. The robustness of the results with respect to both the value chosen for the dielectric constant and the discretization parameters was tested; the results are given in the Supporting Information. The QM part was described using the same methods as in the reference paper:^25^ TDCAM-B3LYP/6-31+G(d) for excitation energy calculations and PBE0/6-31G(d) for geometry optimizations.

Excitation energy calculations were performed using the modified Gaussian 16 suite,^35^ whereas geometry optimizations were performed using the interface^38,40^ between Tinker^36,37^ and the modified Gaussian 16 suite.

In the geometry optimizations, we kept frozen all the MM residues except those directly linked to the QM part.

### 3.2 Timings

As a preliminary analysis, we report on the performance of the new three-layer model, compared with the reference two-layer model. For these tests, we used  = 2 *N*_grid_ = 26, as this choice reproduces the same results obtained with finer discretizations, and spherical harmonics of at least maximum angular momentum of 2 are required to properly treat the AMOEBA quadrupoles. A benchmark of the results against the discretization is provided in the Supporting Information. All the calculations have been performed on **mTFP** neutral structures of increasing size.

**Figure 3:**
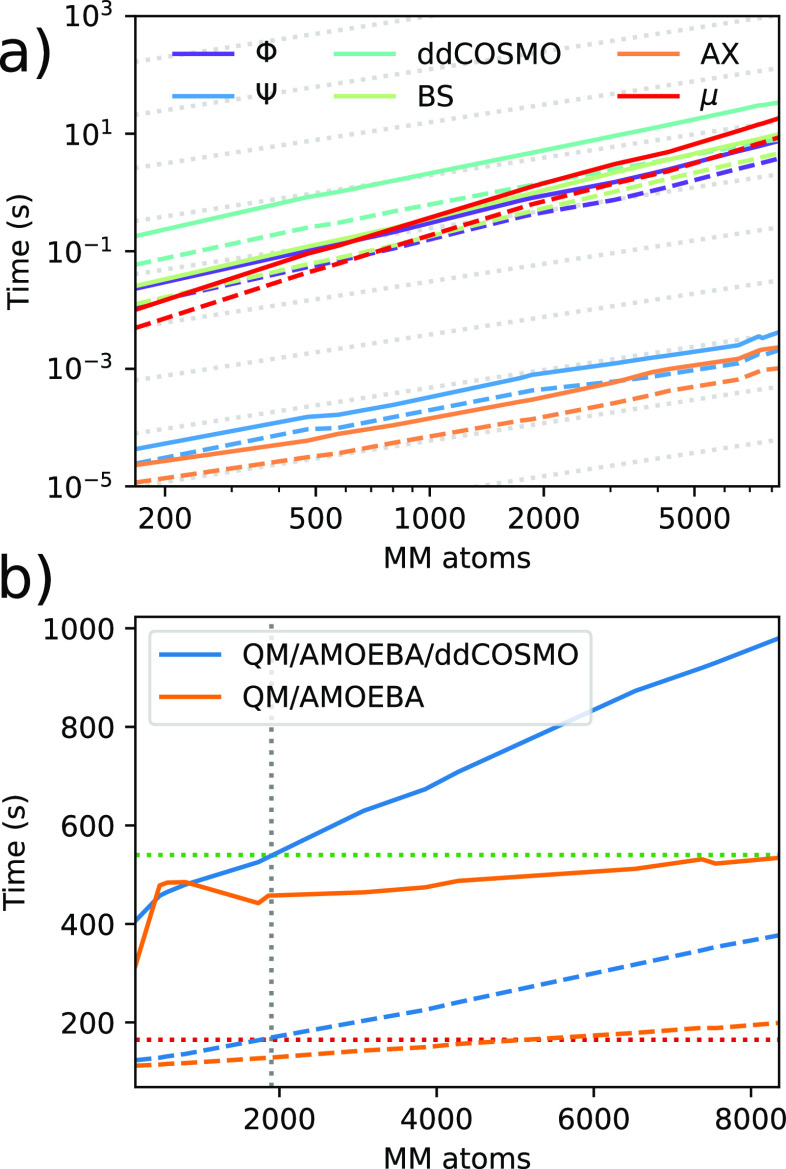
(a): Time required by the various steps reported in algorithm 1, summed over the first SCF iteration (solid lines) and averaged over all SCF iterations (dashed lines). These results are obtained on **mTFP** neutral structures of increasing size. The gray dotted lines report a slope of 1 in the log–log plot. (b) Total time required for a TDDFT calculation (solid lines) and a GS forces calculation (dashed lines) done for a QM/AMOEBA/ddCOSMO and a QM/AMOEBA model. Also in this case, the results are obtained on **mTFP**. To aid the comparison between the two- and three-layer models, the shell radius at which we observed convergence with QM/AMOEBA/ddCOSMO (10 Å) is marked with a dotted gray vertical line. The corresponding QM/AMOEBA/ddCOSMO timing is marked with a dotted green line for the TD-DFT calculation and with a dotted red line for the forces.

Figure 3A reports the timings required for the various steps of the algorithm 1, measured at the first SCF iteration and the average on every SCF iteration. As anticipated, all the steps are linear scaling, with the exception of ddCOSMO which shows a slope slightly greater than 1. The local functions Ψ(μ) and its adjoint (*AX*) are cheap and linear scaling and indeed even for the largest system only require 1 ms. All the electrostatic terms (μ, Φ, and *BS*) are in principle quadratically scaling; however, the FMM implementation effectively allows one to compute them in a linear scaling regime, and for the largest system, they require ∼1 s. Finally, ddCOSMO is the most costly step (due to the microiterations): solving the linear system multiple times requires ∼10 s for the largest system. The cost of each ddCOSMO microiteration is linear; however, because the number of microiterations depends on the system’s size, ddCOSMO exhibits a slightly more-than-linear scaling. A plot of the number of ddCOSMO microiterations required for different shell radii is reported in the Supporting Information.

To further improve the overall performances, we use the solutions of the previous iteration as guesses for both the macro- and microiterations. For this reason, in Figure 3A, the average timings are smaller with respect to those measured at the first SCF iteration. Reducing the number of macroiterations reduces the average cost of all the steps; furthermore, reducing the number of microiteration further reduces the average cost of the ddCOSMO step.

Figure 3B reports the total time required for a ground state (GS) forces calculation and a TDDFT calculation on **mTFP** neutral structures. As expected, the more complex three-layer model results in a more time-consuming calculation with respect to the simpler two-layer one.

However, as we shall show in the following section, three-layer calculations provide accurate results even on smaller radii (<10 Å), whereas in the case of a two-layer calculation, a much larger radius is required (∼15 Å for **mTFP** and **Dronpa**, ∼20 Å for **PhiYFP**). By considering these two radii, we observe that the two investigated models result in calculations with a similar computational cost for the GS forces calculation, whereas for the excited state calculation, the three-layer model is still slightly more expensive.

### 3.3 Electrostatics and Polarization

**Figure 4:**
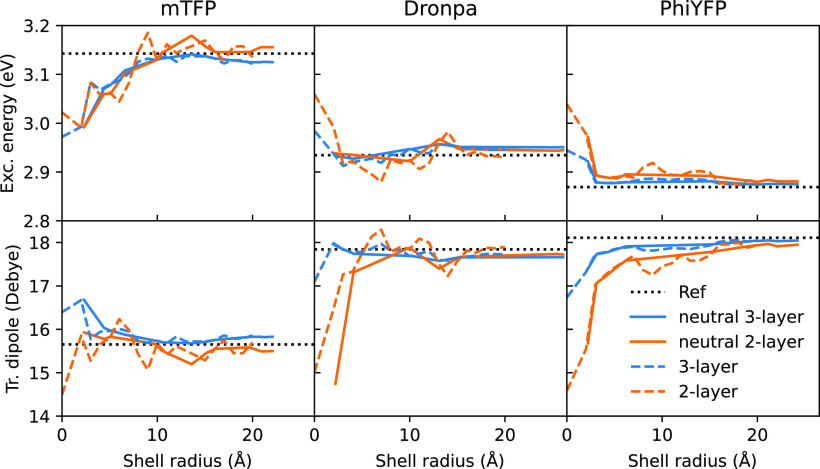
First excited-state properties for the three GFPs at different shell radii. The calculations were performed using both a QM/AMOEBA/ddCOSMO and a QM/AMOEBA model. Calculations on neutral structures are reported using solid lines, whereas calculations done on generic structures are reported using dashed lines. The reference values, computed using QM/AMOEBA on the complete systems, are reported as dotted lines. Values at a shell radius of 0 Å are computed without MM atoms, so that the two models correspond to QM/ddCOSMO and QM models, respectively.

In this section, we report a comparison of the excitation energies computed using three-layer and two-layer models on the three GFPs using model systems of increasing size (either obtained with the strategy which gives neutral systems or with the general one). The transition energies and transition dipoles calculated for the first excited state are reported in Figure 4.

Considering first the results computed on neutral systems, we observe that, for the three GFPs, the three-layer model converges faster for both excitation energy and transition dipole moment. Moreover, the three-layer model damps the oscillations of the properties which are present in the results obtained using the two-layer model. A shell radius of ∼5 Å in combination with the three-layer model is enough to compute converged properties for **Dronpa** and **PhiYFP**. On the other hand, properties of **mTFP** converge only with a radius of ∼9 Å. This larger radius is required because of the peculiar arrangement of charged residues close to **mTFP** chromophore. Using the two-layer model, larger shell radii (15–20 Å) are necessary to obtain converged results. Similar findings are also found in the literature.^9,12^

Considering the results of all (neutral and charged) systems, we observe much larger oscillations in the properties computed using the two-layer model. These oscillations directly stem from the unbalanced charge of the systems. However, switching to a three-layer description greatly damps the oscillations, and the obtained results do not differ significantly with respect to those computed on neutral systems only.

To assess the role of polarization, we compared the results of three-layer and two-layer calculations based on AMOEBA, with results obtained using QM/MM/ddCOSMO, QM/MM, QM, and QM/ddCOSMO. In this comparison, we limited the analysis to neutral systems only. Due to the high computational cost, QM (and QM/ddCOSMO) calculations were performed only for the smallest systems.

**Figure 5:**
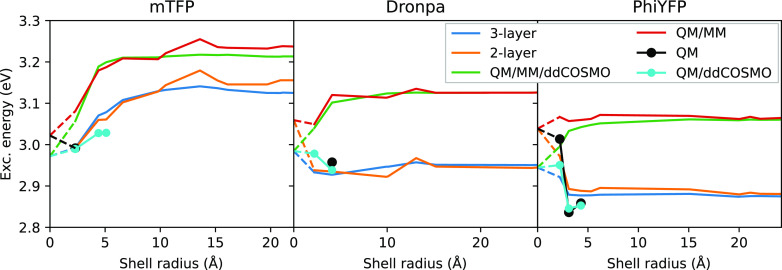
Excitation energy computed using polarizable, nonpolarizable, and full QM models. The values at a shell radius of 0 Å report results computed on systems not containing MM atoms, corresponding to QM/ddCOSMO and QM/vacuum cases. The black and cyan data report results obtained using a QM description for all the atoms of the structure and in the cyan case in combination with ddCOSMO to improve TDDFT results.

Figure 5 shows the excitation energy on the three GFPs for the various models. First, we note that the effect of adding a third continuum shell is similar for both AMOEBA and the nonpolarizable MM description. In detail, we observe the same deviations of the two-layer profile with respect to the three-layer profile for both the polarizable and nonpolarizable cases.

When instead we compare AMOEBA and the nonpolarizable MM, we see important differences. In particular, for **Dronpa** and **PhiYFP**, we observe that a nonpolarizable environment blue-shifts the excitation energy, whereas a polarizable environment red-shifts it with respect to the vacuum. The addition of a third continuum shell partially compensates for the lack of polarization in the atomistic MM shell; this effect, however, is gradually lost when the shell radius increases, until convergence with the QM/MM result.

Finally, we compared the results with the excitation energies obtained from full QM calculations (possible only on the smallest shells). The results show an almost quantitative agreement between the AMOEBA and full QM results, whereas with the nonpolarizable embedding, the agreement is lost. Also, in the **mTFP** case, where both the polarizable and nonpolarizable embeddings are qualitatively similar, the AMOEBA results are quantitatively closer to the QM results.

We also considered the case of three-layer and two-layer calculations in which the AMOEBA polarizabilities are non-zero only in a fixed shell around the QM region. The idea is to recover the effect of the fully polarizable AMOEBA embedding with a reduced computational cost. This analysis, however, showed that the partially polarizable AMOEBA shell does not lower significantly the computational cost neither for two-layer nor for three-layer calculations. For this reason, the results and the analysis are only reported in the Supporting Information.

### 3.4 Comparison with the Experiments

As a last analysis, we investigated the role of the three-layer model on geometrical properties and we compared the excitation energies of the optimized structures with experiments. We performed a series of geometry optimizations on the neutral structures using the QM/AMOEBA/ddCOSMO model. Both the optimization and excited-state calculations were repeated using the two-layer model.

**Figure 6:**
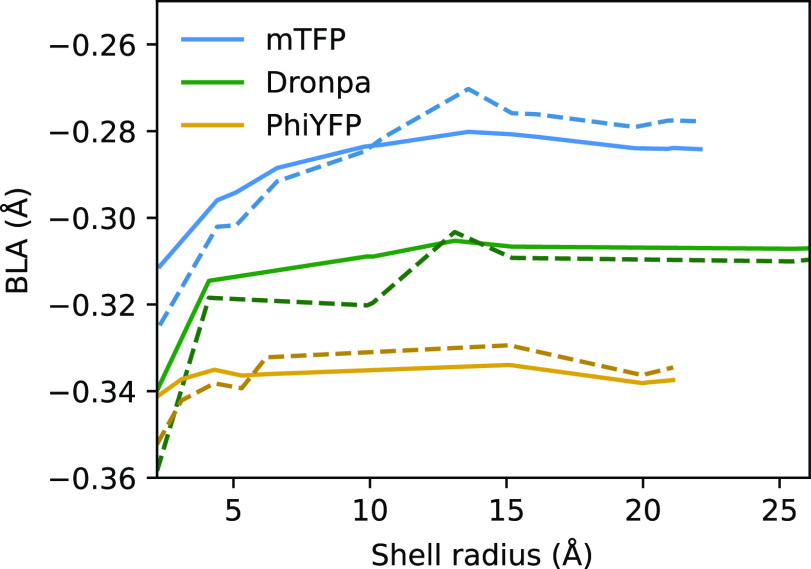
BLA computed on optimized geometries. The results obtained using QM/AMOEBA/ddCOSMO are reported as solid lines, whereas those obtained using QM/AMOEBA are reported as dashed lines.

To compare the optimized geometries, we measured the bond length alternation (BLA) using a definition obtained from a principal component analysis done in a previous work:^53^ its full expression is provided in the Supporting Information. A plot of the BLA values obtained for different shell radii is provided in Figure 6 for the three GFPs. For **Dronpa** and **PhiYFP**, both the two-layer and three-layer descriptions converge to a similar value, whereas for **mTFP**, we observe a small discrepancy. In all the cases, the three-layer model shows smaller fluctuations compared to the two-layer model, and it converges faster.

**Figure 7:**
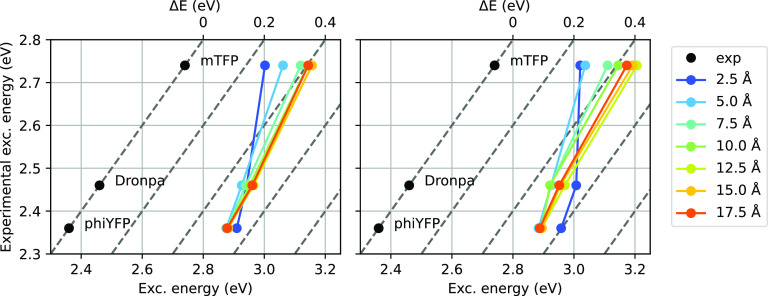
Experimental excitation energies plotted against computed ones, left: QM/AMOEBA/ddCOSMO, right: QM/AMOEBA. Values computed at different shell radii are reported using different colors. The plot format is intended to highlight both the absolute shift with respect to experimental values and the relative differences between different GFPs. A perfect relative agreement corresponds to a slope of 1, which is indicated by dashed gray lines.

A comparison of the excitation energies computed using the two-layer and three-layer models against experimental values is reported in Figure 7. We observe that the TDDFT/AMOEBA description systematically overestimates excitation energies by ∼0.4 eV. To further improve this result, it is necessary to go beyond TDDFT, as shown in ref (25). The description provided by the three-layer and two-layer methods is similar, provided that a large enough MM shell is used, with the only substantial difference being the **mTFP** excitation energy, which is slightly lower in the case of the three-layer method. This discrepancy arises due to the different BLA of the optimized structures.

The higher sensitivity of the **mTFP** system to the embedding model (in terms of both the shell radii and the two- vs three-layer coupling) is due to its larger response to the electrostatic field in the chromophore cavity,^25,46^ which in turn can be explained by the larger driving force between the two chromophore resonance forms.^54^ By contrast, the smaller driving force in **PhiYFP** results in much reduced sensitivity and hence faster convergence with the shell size. Overall, this system-dependent sensitivity to the environment highlights the importance of carefully taking into account the electrostatic effects at both a short and a long range. The three-layer approach describes both effects in a balanced and reliable way already with relatively small embeddings.

## 4 Conclusions

We presented the theory and implementation of a fully polarizable three-layer QM/MM/continuum approach, which combines the AMOEBA force field and ddCOSMO. The implementation can be used to perform ground-state SCF energies and forces and linear response excited-state calculations. The extension to excited-state forces will be the subject of future development.

As a test case, we investigated the excitation energies of three variants of the GFP by comparing the three-layer approach with the corresponding two-layer QM/AMOEBA, and both a three-layer and two-layer model based on a nonpolarizable AMBER force field. For all the three systems, the use of a polarizable force field is necessary not only to improve the quantitative agreement with a full QM calculation, but also to achieve a correct qualitative estimation of the effects due to the protein. The effect of the third continuum shell is instead similar on both descriptions, with the exception that, for small-size systems, it compensates for the lack of polarization in the MM shell. Moreover, the third continuum shell leads to a faster convergence of the results against the size of the MM shell, especially in the case of charged structures. This lower sensitivity to the charge dispenses with the need of selecting neutral subsystems and is useful, for example, when averaging over the snapshots of a MD simulations, where charged residues and ions can easily go in and out of a defined shell.

To conclude, the QM/AMOEBA/ddCOSMO model appears to be the best choice in all cases where only small and/or non-neutral systems are available. In the other cases, its application leads to results similar to a QM/AMOEBA calculation at a comparable, or sometimes slightly higher, computational cost. We believe that further investigation and the development of new strategies to reduce the cost of the three-layer model are therefore paramount to make it more generally applicable. From this point of view, we believe that one of the main issues of the present implementation is that the molecular cavity used by ddCOSMO uses one sphere per atom. Especially for calculations involving large MM embeddings, this is certainly not an efficient choice. Simplifying the cavity, adopting for instance a coarse-grained definition where only heavy atoms or even the whole residues are associated with spheres, can introduce major savings in the three-layer model. A careful optimization of the radii of the spheres can also ensure that the quality of the results is not spoiled by the cavity’s simplification. The implementation of ddCOSMO, and therefore of the QM/AMOEBA/ddCOSMO model, using a more general molecular cavity is the subject of active investigation.

## Supporting Information Available

Information about the neutral structures of increasing size; representations of the neutral structures of increasing size; BLA definition; computational cost analysis for partially polarizable description; and additional plots (PDF)
