# Supplementary material for: Energy, Structures, and Response Properties with a Fully Coupled QM/AMOEBA/ddCOSMO Implementation
Source: J Chem Theory Comput. 2021 Sep 3;17(9):5661–72. doi: 10.1021/acs.jctc.1c00555 (PMC8444335; doi:10.1021/acs.jctc.1c00555)
Supplement: Supplementary file 1 — ct1c00555_si_001.pdf [file ct1c00555_si_001.pdf]

Supporting Information: A fully polarizable QM/MM/continuum implementation

Michele Nottoli,*,† Riccardo Nifosì,‡ Benedetta Mennucci,† and Filippo Lipparini†

†*Dipartimento di Chimica e Chimica Industriale, Università di Pisa, Via G. Moruzzi 13, I-56124 Pisa, Italy*

‡*NEST, Istituto Nanoscienze-CNR and Scuola Normale Superiore, Piazza San Silvestro 12, I-56127 Pisa, Italy*

E-mail: michele.nottoli@phd.unipi.it

Information about the neutral structures of increasing size

Table 1: Shell radius (r), number of MM atoms and fraction of protein included in the MM part (f) for each neutral structure used in the analysis. The cases reported in parenthesis are only used for partially polarizable or non polarizable calculations.

**mTFP**			**Dronpa**			**PhiYFP**		
r (Å)	MM Atoms	f	r (Å)	MM Atoms	f	r (Å)	MM Atoms	f
2.3	167	0.05	2.2	167	0.05	2.2	123	0.03
4.4	476	0.13	4.1	413	0.11	3.1	329	0.08
5.1	573	0.15	9.9	1173	0.45	4.3	409	0.10
6.6	803	0.21	10.1	1819	0.46	5.3	530	0.13
9.8	1732	0.44	13.1	2988	0.67	6.2	719	0.19
10.5	1860	0.47	15.2	3886	0.79	15.1	3667	0.72
13.6	3071	0.68	25.5	11574	1.00	20.0	6855	0.95
15.2	3855	0.78	26.1	12195	1.00	20.1	6879	0.95
16.1	4274	0.83	(27.1	13369	1.00)	21.1	7671	0.97
19.7	6531	0.96	(28.1	14578	1.00)	22.1	8520	0.99
20.9	7376	0.99	(29.1	15919	1.00)	24.2	10732	1.00
21.1	7550	0.99	(30.1	17296	1.00)	(25.2	11786	1.00)
—	—	—	(36.0	26456	1.00)	(26.1	12809	1.00)
—	—	—	(36.1	26597	1.00)	(33.9	24587	1.00)
—	—	—	(38.0	29011	1.00)	(34.1	24821	1.00)
—	—	—	(38.1	29098	1.00)	—		

Representations of the neutral structures of increasing size

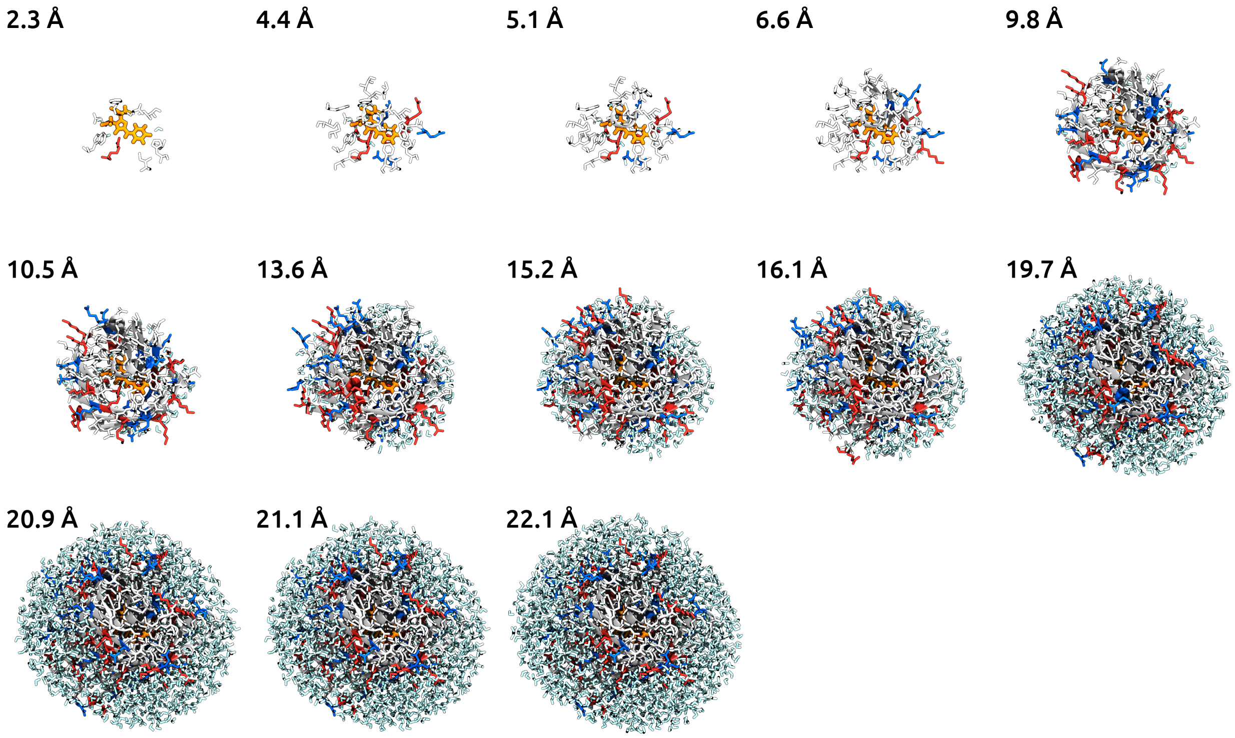

Figure 1: Representations of neutral cropped structures of **mTFP**. The QM residue is drawn in orange, positively charged residues are drawn in red and negatively charged residues are drawn in blue and solvent molecules are drawn in cyan.

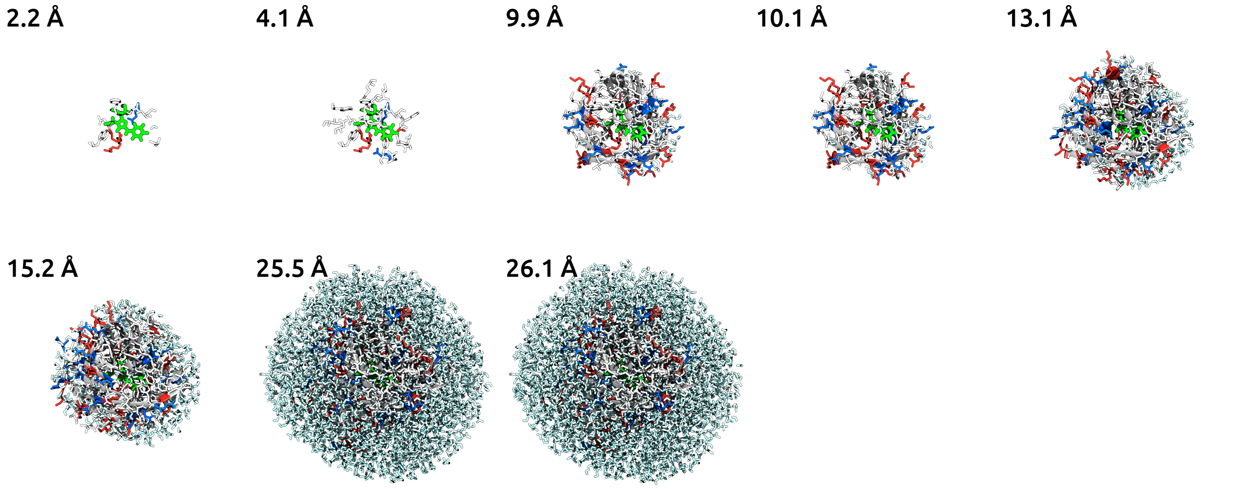

Figure 2: Representations of neutral cropped structures of **Dronpa**. The QM residue is drawn in green, positively charged residues are drawn in red and negatively charged residues are drawn in blue and solvent molecules are drawn in cyan.

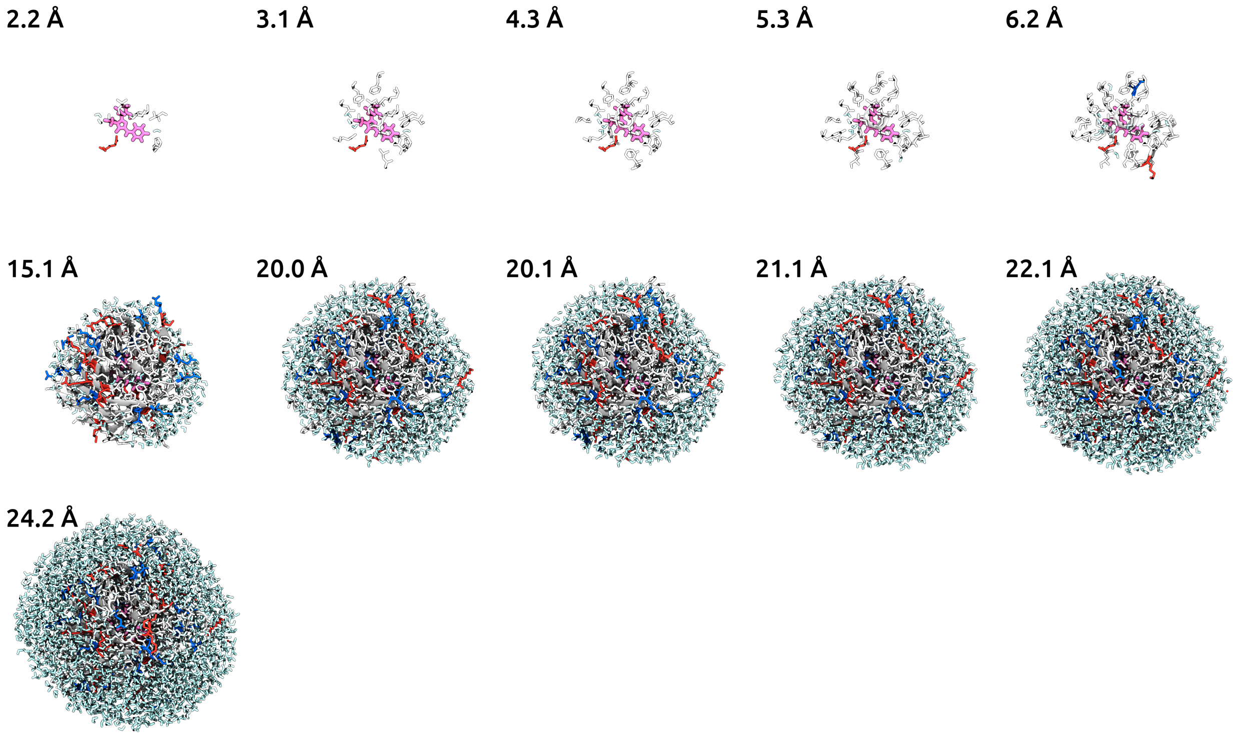

Figure 3: Representations of neutral cropped structures of **PhiYFP**. The QM residue is drawn in purple, positively charged residues are drawn in red and negatively charged residues are drawn in blue and solvent molecules are drawn in cyan.

Bond length alternation definition

Table 2: The bond length alternation is defined as a linear combination of bond distances. The table and the figure provide the coefficients and the atom definitions.

Coefficient	Atom 1	Atom 2
0.32	OH	CZ
-0.27	CZ	CE2
-0.27	CE1	CZ
0.17	CE2	CD2
0.17	CD1	CE1
-0.24	CD2	CG2
-0.24	CG2	CD1
0.38	CG2	CB2
-0.39	CB2	CA2
0.32	CA2	C2
0.02	C2	O2
-0.34	C2	N3
0.21	N3	C1
-0.08	C1	N2
0.05	N2	CA2

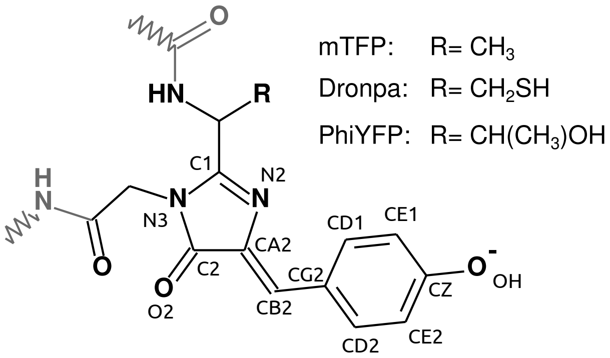

Partially polarizable AMOEBA descriptions

To further assess the role of polarization, we repeated 3-layer and 2-layer calculations on the neutral structures, by setting non zero polarizabilities only in part of the AMOEBA shell. We tested the fully non polarizable case, and the cases with a polarizable sub-shell close to the QM part of 5, 10 and 15 Å. We then compared the results against the fully polarizable case and a full QM/ddCOSMO description. Due to the high computational cost QM/ddCOSMO calculations were performed only for the smallest systems.

Figure 4 reports the excitation energy on the three GFPs for the various models. First, we note that the effect of adding a third continuum shell is similar for both the fully polarizable case, the partially polarizable cases and the non polarizable description. We observe the same deviations of the 2-layer profile with respect to the 3-layer profile regardless of the size of the polarizable sub-shell. The role of polarization in the MM layer is, on the other

hand, much more significant. The correct qualitative behavior is already recovered with a polarizable sub-shell of just 5 Å, however, for the **mTFP** and **PhiYFP** systems, a larger polarizable sub-shell (>15 Å) is needed to recover quantitatively the description obtained with a fully polarizable model.

We conclude this discussion with a computational consideration: a plot of the timings obtained with a partially polarizable AMOEBA embedding is reported in the figure 5. Using a partially polarizable description has a sizeable impact on the computational cost only if a very small polarizable radius can be used and only on calculations performed using the 3-layer model, as can be deduced from the timings reported in the supporting information. In fact, reducing the size of the polarizable layer mainly affects the calculation of the coupling terms, while the overall cost of the calculation is dominated by ddCOSMO. Given that converged results are only obtained for larger polarizable shells, using a hybrid polarizable/non-polarizable scheme does not seem to be advantageous.

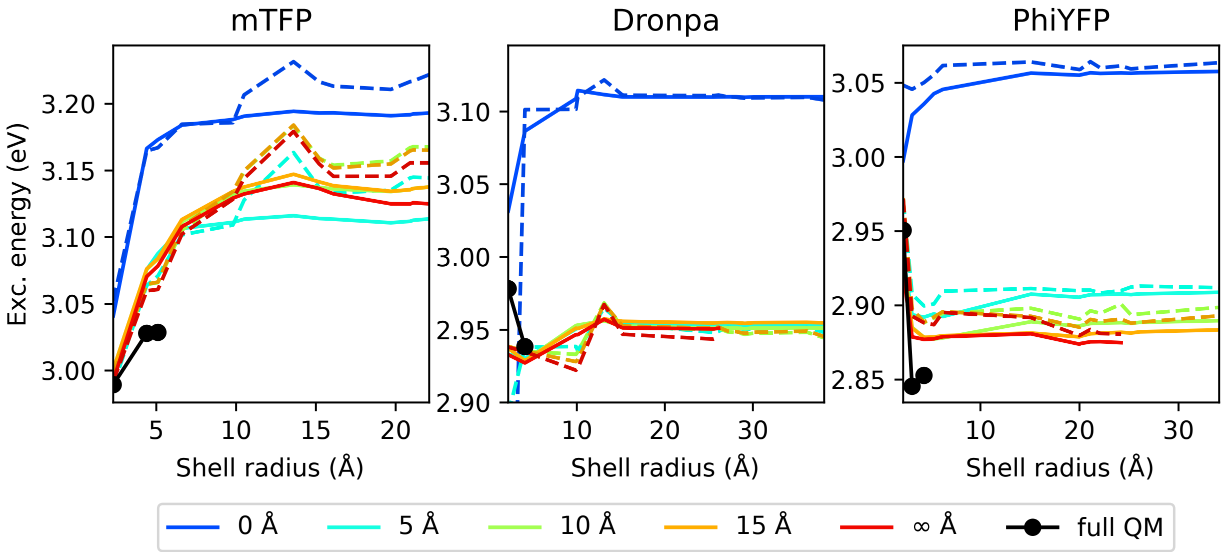

Figure 4: Excitation energies computed using polarizable, partially polarizable, non polarizable and full QM models. The colored lines report results obtained by including polarization in shells of increasing size, going from 0 Å to a completely polarizable description (∞ Å). Solid lines report results obtained using the 3-layer model, dashed lines report results obtained using the 2-layer model. The black lines report results obtained using a QM description for all the atoms of the structure, in combination with ddCOSMO to improve TDDFT convergence.

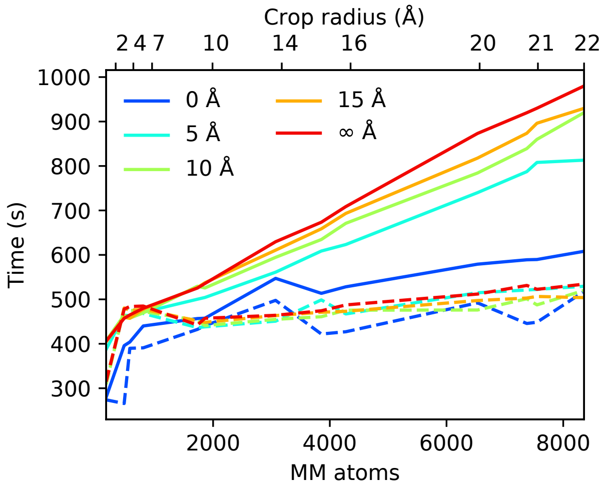

Figure 5: Timings for 3-layer and 2-layer calculation done on **mTFP** systems with varying AMOEBA shell radii (x axis) and varying polarizable AMOEBA sub-shell radii (different colored lines). The ∞ Å corresponds to a fully polarizable description. Solid and dashed lines report 3-layer and 2-layer results respectively.

Additional plots

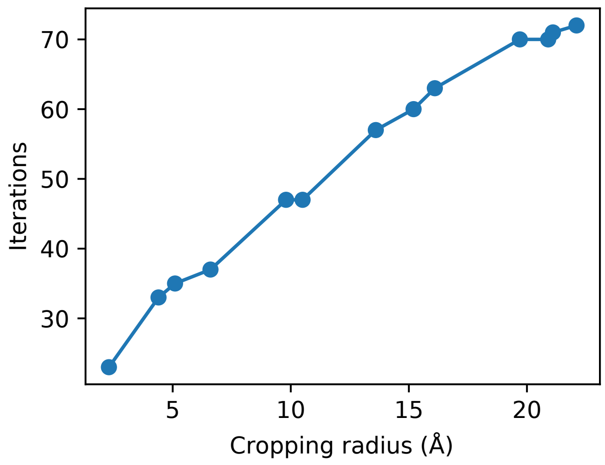

Figure 6: Iterations required for the first occurrence of the ddCOSMO linear system. The calculations are done on neutral cropped structures of **mTFP**.

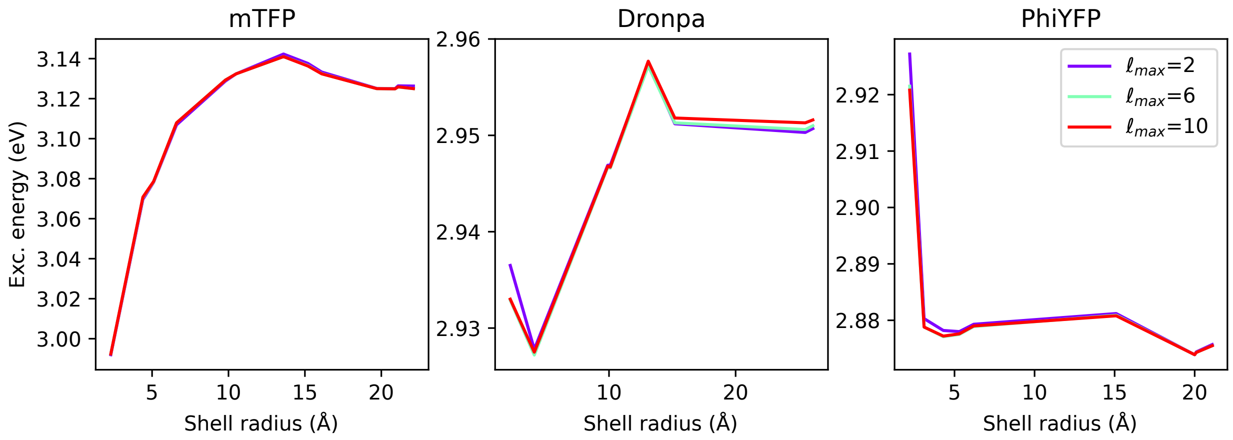

Figure 7: Effect of different discretizations on the excitation energies computed on the neutral structures of the three systems.

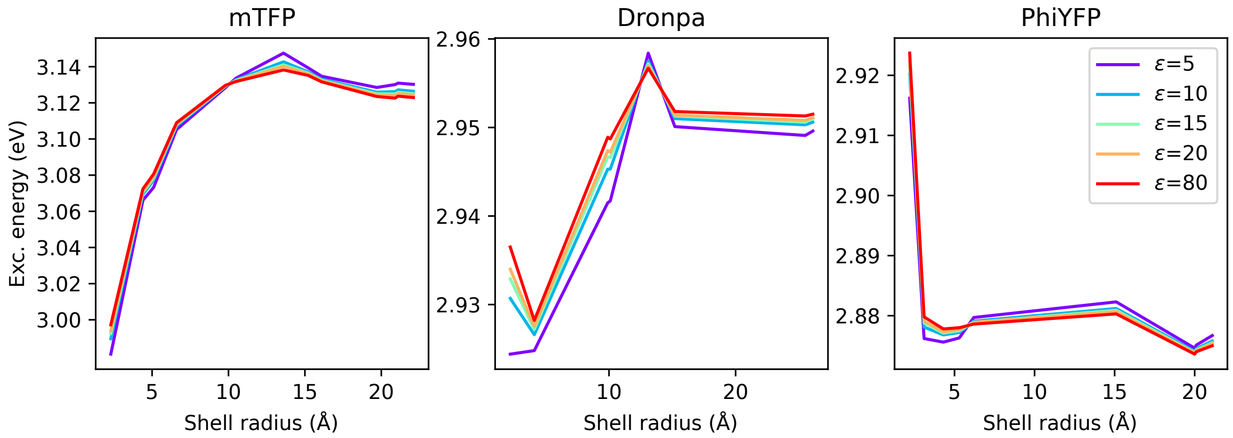

Figure 8: Effect of different dielectric constants on the excitation energies computed on the neutral structures of the three systems.
